# Occurrence, antifungal susceptibility, and virulence factors of opportunistic yeasts isolated from Brazilian beaches

**DOI:** 10.1590/0074-02760180566

**Published:** 2019-03-14

**Authors:** Natália OP Maciel, Susana Johann, Luciana R Brandão, Sona Kucharíková, Camila G Morais, Alexandre P Oliveira, Gustavo JC Freitas, Beatriz M Borelli, Franciane M Pellizzari, Daniel A Santos, Patrick Van Dijck, Carlos A Rosa

**Affiliations:** 1 Universidade Federal de Minas Gerais, Instituto de Ciências Biológicas, Departamento de Microbiologia, Belo Horizonte, MG, Brasil; 2 VIB-KU Leuven Centre for Microbiology, Leuven, Belgium; 3 Institute of Botany and Microbiology, KU Leuven Laboratory of Molecular Cell Biology, Leuven, Belgium; 4 Universidade Estadual do Paraná, Laboratório de Ficologia e Qualidade de Água Marinha, Curitiba, PR, Brasil

**Keywords:** opportunistic pathogenic yeasts, beaches, Escherichia coli, Candida albicans, yeast adhesion, disseminated infection tests

## Abstract

**BACKGROUND:**

Opportunistic pathogenic yeast species are frequently associated with water habitats that have pollution sources of human or animal origin. *Candida albicans* has already been suggested as a faecal indicator microorganism for aquatic environments.

**OBJECTIVES:**

The goal of this study was to investigate the occurrence of *C. albicans* and other opportunistic yeasts in sand and seawater samples from beaches in Brazil to assess their correlation with *Escherichia coli*, and to characterise the pathogenic potential of the yeast isolates.

**METHODS:**

Opportunistic species (yeasts that grow at 37ºC) were isolated from sand and seawater samples from eight beaches in Brazil during the summer and the winter. Opportunistic yeast species were evaluated for their susceptibility to antifungal drugs, virulence factors, and the *in vitro* and *in vivo* biofilm formation. Strains were selected to carry out virulence tests using BALB/c mice.

**FINDINGS:**

Several water samples could be classified as inappropriate for primary contact recreation in relation to *E. coli* densities. *C. albicans* was isolated in low densities. Of the 144 opportunistic yeasts evaluated, 61% displayed resistance or dose-dependent sensitivity to at least one tested drug, and 40% produced proteinase. Strains of *C. albicans* and *Kodamaea ohmeri* exhibited the highest rates of adhesion to buccal epithelial cells. All the *C. albicans* strains that were tested were able to undergo morphogenesis and form a biofilm on catheter fragments in both *in vitro* and *in vivo* experiments. It was possible to confirm the pathogenic potential of three of these strains during the disseminated infection test.

**MAIN CONCLUSIONS:**

The identification of opportunistic yeast species in seawater and sand samples from Brazilian beaches suggest a potential risk to the health of people who use these environments for recreational purposes.

Beaches rank high among recreational areas worldwide, and many of them are located next to urban areas, where the anthropic pressure is high and, consequently, notable impacts on their physico-chemical and biological characteristics are observed.^1^ Urban development often results in high microbial deposition on beaches. Major threats to coastal waters include municipal sewage discharge, industrial discharge, surface runoff, agricultural endeavors, domestic animals, human bather shedding, and ineffective wastewater treatment. Because most leisure activities of residents and tourists from the coastal areas involve contact with seawater and sand, there is a growing health concern related to the exposure of bathers to the microorganisms present. In recent years, several epidemiological studies revealed a positive correlation between swimming at beaches affected by human activities and symptoms such as gastrointestinal and dermatological diseases, but also respiratory, eye, nose, and throat infections.^2^ ^,^ ^3^ The disease incidence is dependent on several factors such as the extent of pollution, the time and type of exposure, and the immune status of users. Children, for example, may be at a greater risk of illness following such exposures. These effects might be due to differences in immunity or differing behavioral factors such as poor hygiene, longer exposure to, and greater quantities of ingestion of potentially contaminated water and sand.^4^

In relation to beach sand, little is known about the microbial structure in this substrate, and the health implications of the allochthonous microbes introduced in this recreational environment. Allochthonous microbes may include faecal bacteria and pathogens derived from sewage, storm water runoff, or feces from humans or domestic and wild animals.^3^ Most epidemiological studies at recreational beaches have focused on measuring the human health risks associated with exposure to beach water rather than beach sand, despite the fact that people tend to spend a majority of their time in contact with the sand. In addition, characterisation of the virulence characteristics of putative pathogens detected in beach sand has rarely been done.^3^

For years, faecal indicator bacteria (typically coliforms, *Escherichia coli*, as well as faecal *Streptococci* and *Enterococci*) have been used to assess the water and sand quality of beaches. Their association with diseases is often described in areas with known sources of pollution.^5^ However, it has been shown the persistence and growth of these microorganisms in the environment, which is an undesirable feature for a good faecal indicator.^3^ Environmental persistence of faecal indicator bacteria compromises their utility in recreational water quality monitoring because the presence of these organisms would not necessarily indicate a recent contamination event, and in some cases, could lead to an overestimation of the associated public health risk.^6^ Additionally, it has been recognised that faecal bacterial indicators are not necessarily good predictors of the presence of important pathogens such as enteroviruses, protozoa, and fungi.^1^ ^,^ ^6^

In addition to the traditionally used indicators, other microbiological parameters could be adopted to improve water and sand quality evaluation. Yeasts are an alternative, because these microorganisms represent a widely distributed group that is easily cultivated and has a well-developed taxonomy. Yeast diversity and density in aquatic environments may be influenced by the presence of allochthonous sources such as soil, plant debris, and sewage, as well as by the pH, temperature, and UV radiation.^7^ Several *Candida* species of the *C. albicans*/*Lodderomyces* clade, for example, are frequently associated with water habitats that have pollution sources of human or animal origin. Furthermore, *C. albicans* has already been suggested as a faecal indicator microorganism for aquatic environments.^8^ Based on the possibility that yeasts may represent a potential risk to beach users, we determined the occurrence of opportunistic species (yeasts that grow at 37ºC) in sand and seawater samples from eight beaches in Brazil and evaluated their correlation with *E. coli*, one of the conventional indicators of faecal contamination. Furthermore, the pathogenic potential of the yeast isolates was assessed by the characterisation of virulence factors, antifungal susceptibility, and the ability to cause disease in a murine model.

## MATERIALS AND METHODS

*Sampling areas* - Sand and seawater samples were collected from two recreational beaches in the city of Rio de Janeiro, Rio de Janeiro state, Brazil: Ipanema (22º59’12.6”S 43º12’11.8”W) and Copacabana (22º58’32.1”S 43º11’13.6”W); and six recreational beaches from the Paraná coast, Southern Brazil: Praia de Leste (25º42’08.1”S 48º28’14.5”W), Ipanema balneary (25º39’25.2”S 48º26’30.8”W), and Shangrilá balneary (25º37’36.3”S 48º25’07.1”W), in the municipality of Pontal do Paraná, Florida balneary (25º46’48.1”S 48º30’57.2”W), Praia Central de Matinhos (25º48’49.1”S 48º31’57.1”W), and Praia Mansa (25º51’02.1”S 48º32’49.0”W), in the municipality of Matinhos.

*Sampling* - Three sampling points were chosen for each beach in Rio de Janeiro and one point for each beach in Paraná. At each point, two transects, running perpendicular to the coastline, were determined. The first transect was 50 m to the north of the mouth of a storm drainage system, and the second one was 50 m to the south of it. The seawater samples of 400 mL were collected during the summer (in January 2010 at the Paraná beaches and in February 2011 at the Rio de Janeiro beaches) and the winter (in July 2010 for Paraná and in August 2010 for Rio de Janeiro). Sand samples were collected at three different zones along the transects: supralittoral, mediolittoral, and infralittoral.^9^ ^,^ ^10^ Each sample of 100 g was a composite of the top 10 cm of sand from an area of 0.25 m^2^. Sand samples were placed in sterile plastic bags and transported to the laboratory on ice. The samples were processed within 24 h of collection.

One sample of superficial seawater was collected at each transect and one in front of the mouth of the storm drainage system. Samplings were performed at places where the water depth was around 1 m. Water samples were transported to the laboratory in sterile flasks on ice and processed within 24 h of collection. The temperature of each water sample was measured at the time of sampling using a digital thermometer. Salinity and pH were measured for each water sample at the laboratory using a refractometer and a pH meter, respectively.

*Yeast isolation* - Yeast isolation was performed using the membrane filtration method and three different culture media. Yeast extract-Malt extract agar (YM: 0.5% peptone, 0.3% yeast extract, 0.3% malt extract, 1% glucose, 2% agar, and 20 mg% chloramphenicol) was used to determine the total yeast counts. CHROMagar Candida (Difco, Sparks, USA) was used for the differential isolation of opportunistic yeast species. mCA agar was used for the selective and differential isolation of *C. albicans* strains, as described by Buck and Bubucis.^8^ Twenty-five grams of sand was added to 200 mL of sterile phosphate-buffered saline (PBS) and shaken vigorously for 1 min.^1^ Ten milliliters of each sand suspension and 50 mL of each water sample were filtered through 0.45 μm sterile membrane filters (Millipore, Cork, Ireland), which were then placed onto the culture media. YM agar plates were incubated at 25ºC and plates of CHROMagar Candida and mCA agar at 37ºC for 3-7 days. After incubation, yeast colonies were counted and the yeast density was expressed as the number of colony-forming units (CFU) per 100 mL for the water samples or per gram for the sand samples.

Yeast colonies growing on CHROMagar Candida and mCA agar plates were grouped based on their colour, texture, brightness, shape, and size. Representatives of each different morphotype were picked from the plates and pure cultures were obtained. Yeast cultures were preserved in GYMP broth (glucose-yeast extract-malt extract-peptone broth: 2% glucose, 0.5% yeast extract, 1% malt extract, and 0.2% potassium phosphate dibasic) and 15% glycerol at -80ºC.

*Yeast identification* - Yeast isolates with similar morphological characteristics were grouped and subjected to polymerase chain reaction (PCR) fingerprinting using the intron splice-site primer EI-1.^11^ Isolates with identical DNA-banding patterns were considered to putatively belong to the same species. At least 50% of the yeast isolates of each molecular group were identified by sequencing. Species identification was performed by sequence analysis of the ITS-5.8S region and the D1/D2 variable domains of the large subunits of rRNA genes, as described previously.^12^ The samples were sequenced by the capillary electrophoresis apparatus ABI3130, using BigDye v3.1 and the POP7 polymer. The sequences obtained were compared with those deposited at the GenBank database (National Center for Biotechnology Information, NCBI), using the Basic Local Alignment Search Tool (BLAST at http://www.ncbi.nlm. nih.gov).

*Escherichia coli quantification* - *E. coli* densities in sand and seawater samples were determined using the substrate technique Colilert (IDEXX, Lenexa, KS, USA). The culture medium was added to 100 mL of seawater or sand suspensions. The samples were mixed by hand, poured onto the trays, and incubated at 36ºC for 24 h. The most probable number (MPN) of *E. coli* in each sample was determined according to the manufacturer’s instructions.

*Antifungal susceptibility testing* - Yeasts belonging to opportunistic pathogenic species were tested for *in vitro* susceptibility to amphotericin B (Sigma-Aldrich, St. Louis, MO, USA), itraconazole (Sigma-Aldrich), and fluconazole (Sigma-Aldrich). The tests were performed according to the broth microdilution method, described in the M27-A3 of the Clinical Laboratory Standards Institute (CLSI),^13^ in Roswell Park Memorial Institute (RPMI) medium. Microtiter plates (96-well) containing inocula and appropriate concentrations of antifungal drugs were incubated at 35ºC, and the minimum inhibitory concentration (MIC) endpoints were read visually 24 and 48 h after incubation. Drug ― and yeast-free controls were included in all experiments. Yeast isolates that had some clinical importance were categorised in accordance with MIC breakpoints established by M27-A3.^13^ For isolates with species-specific clinical breakpoints, M27-S4^14^ was considered. Two reference clinical strains of *C. albicans*, SC5314 and ATCC18804, were also used in the tests.

*Yeast adhesion to buccal epithelial cells* - The adhesion of opportunistic yeasts to buccal epithelial cells (BECs) was evaluated according to the methods described by Kimura and Pearsall.^15^ The number of adherent yeast cells was quantified by light microscopy at 400× magnification. In each experiment, 50 BECs were examined for adherent yeast cells. Clumped, folded, or overlapping BECs were excluded.

*Proteinase activity* - Proteinase activity was evaluated by halo formation on a medium containing bovine serum albumin (BSA). Proteinase activity was scored as “-” when no visible clearing was present, “1” when proteolysis occurred 1-2 mm around the colony, and “2” when agar discoloration largely exceeded the margin of the colony (3-5 mm).

*Morphogenesis survey* - Yeast strains isolated from beaches that were identified as *C. albicans* (UFMG-CM-Y4044, Y4123, Y4228, Y4236, and Y4622), as well as *C. tropicalis* UFMG-CM-Y4335 and *Kodamaea ohmeri* UFMG-CM-Y4141, which were two other opportunistic yeast species isolated in this study, were submitted for additional tests to assess morphogenesis ability and *in vitro* and *in vivo* biofilm formation. Additionally, seven more strains of *C. albicans* (UFMG-CM- Y3447, Y3448, Y3471, Y3472, Y3476, Y3482, and Y3492) isolated from Brazilian freshwater lakes (unpublished data) were included in these experiments to contribute data regarding the virulence factors of environmental strains representing this species. *C. albicans* strain SC5314 was included as a positive control in all tests as it represents a clinical isolate. These yeasts were grown overnight on yeast extract-peptone-dextrose (YPD) agar (bacteriological peptone 2%, yeast extract 1%, glucose 2%, agar 2%) at 28ºC. Next, the yeasts were resuspended in PBS and approximately 10 to 50 cells of each strain were inoculated on different filament-inducing solid media: medium containing foetal calf serum 10% (Sigma, USA), Spider medium, SLAD medium, and Lee’s medium.^16^ Plates were incubated for five days at 30ºC. Cultivation under embedded-growth conditions was performed as well.^17^ These plates were also incubated for five days at 37ºC. Individual colonies of each strain on each medium were photographed. Strains were also tested in liquid YPD medium supplemented with foetal calf serum 10% and visualised through an Olympus FV1000 confocal microscope.

*Biofilm formation in vitro* - *In vitro* biofilm formation assays with the selected opportunistic yeast strains were performed as previously described by Řičicová et al.^18^ Three fragments of serum-coated polyurethane catheters (Arrow International Reading, Reading, USA) were used for each yeast strain tested. The adhesion phase was followed by a 24 h incubation period. The catheter pieces were then washed twice with PBS and sonicated to obtain the biofilm-forming cells. Biofilm quantification was performed by plating dilutions of the cell suspensions (ranging from 0.001 to 0.1) onto YPD agar. Plates were incubated for 48 h at 37ºC and colony-forming unit (CFU) were counted. The adhesion quantification of the strains was also determined by CFU counting after the adhesion phase. The tests were independently repeated thrice. Strains that showed different biofilm formation profiles during the *in vitro* tests in comparison with the reference strain *C. albicans* SC5314 were selected for the *in vivo* tests.

*Biofilm formation in vivo* - *In vivo* biofilms were grown subcutaneously in a murine model as described by Řičicová et al.^18^ Immunosuppressed animals were used as it was previously shown that immunosuppression resulted in a much more reproducible outcome of this type of experiments.^18^ Briefly, immunosuppression was induced in female BALB/c mice (20 g) by the addition of 1 mg/L of dexamethasone to their drinking water. Two animals/time/strain were used for the experiments. Serum-coated polyurethane catheters were challenged with a suspension of 5 x 10^4^ yeast cells (mL)^-1^ for 90 min at 37ºC and, after being washed, were implanted subcutaneously into the lower backs of the mice. Up to six fragments were implanted per animal. After 4 h, 48 h, and six days, mice were euthanised using general anesthesia (ketamine-xylazine solution 80 mg·kg^-1^:15 mg·kg^-1^) followed by cervical dislocation prior to the removal of the catheters. Catheter fragments were washed and sonicated before biofilm quantification by CFU counting. The adhesion quantification of the strains was also determined by CFU counting after the adhesion phase.

*Murine disseminated model* - To determine the virulence of the selected strains, immunocompetent female BALB/c mice (ca. 20 g) were injected with 5 x 10^4^ yeast cells·g^-1^ via the tail vein. Survival was determined and the animal’s weight was monitored every day. For each assay, five mice per yeast strain were utilised. Assays were independently repeated thrice.

*Animals* - Six-week-old female BALB/c mice were obtained from the Biotery Center (CEBIO) of the Institute of Biological Sciences, Universidade Federal de Minas Gerais. The animals were maintained under standard laboratory conditions at a temperature of 25 ± 2ºC and a photoperiod of 12 h. They received standard mouse chow and water ad libitum.

*Statistical analysis* - Data were tested for normality using the Shapiro-Wilk test. Some data were not distributed normally and were thus evaluated by the nonparametric Mann-Whitney U-test. Associations between all measured parameters, i.e. temperature, salinity, pH, *E. coli*, and yeast, were assessed by calculation of Spearman’s correlation coefficients (*r*_s_). Correlations and differences were considered statistically significant when the significance level was 95% (p < 0.05). Survival curves were estimated by the Kaplan-Meier method, and differences among survival curve averages were compared with a Log-Rank test. Differences of at least p < 0.05 were considered significant.

*Ethics* - The use of animals in this study was approved by the Ethics Committee in Animal Experimentation from the Federal University of Minas Gerais (Protocol no. 27/2014).

## RESULTS

Taking both seasons into account, a total of 72 seawater samples and 144 sand samples were collected from the beaches during the study. *E. coli* counts ranged from 2.0 to > 9678.4 MPN·(100 mL)^-1^ in seawater samples and from 0.1 to > 558.5 MPN·g^-1^ in sand samples [Supplementary data (Tables I-II)]. Statistically, there was a significant difference (Mann-Whitney U-test, p < 0.001) between the density of *E. coli* in water samples from Paraná and those from Rio de Janeiro. The *E. coli* densities in the supralittoral zones were statistically different from those in the mediolittoral (U-test, p < 0.001) and infralittoral (U-test, p < 0.001) zones.

Total yeast counts ranged from 0 to 172 CFU·(100 mL)^-1^ in water samples and from 0 to 408.5 CFU·g^-1^ in sand samples [Supplementary data (Tables I-II)]. Comparing the numbers of yeasts in the sand from the three different zones, there were significant differences among each of them (U-test, p < 0.05). The yeasts were prevalent in supralittoral zone with counts ranging from 0.9 to 62 CFU·(100 mL)^-1^. There were no significant correlations between *E. coli* and the yeast densities in either the seawater or the sand samples (p > 0.05).

The water temperature of the Rio de Janeiro beaches was around 23ºC in winter and 25ºC in summer. In Paraná beaches, the water temperature was around 21.5ºC in winter and 26ºC in summer. The total yeast counts were positively correlated with water temperature (Spearman’s correlation, *r*_s_ = 0.296, p < 0.05), whereas no statistical correlation was found between temperature and *E. coli* (*r*_s_ = -0.127, p = 0.323). Seawater samples from Rio de Janeiro had a salinity of 38% during the summer and 35% during the winter. The salinity values from the beaches of Paraná were 34% and 32% during the summer and winter, respectively. All water samples had a slightly alkaline pH, varying from 7.5 to 8.3. There was a positive correlation between *E. coli* and the salinity (*r*_s_ = 0.329, p < 0.05) and a negative correlation with pH (*r*_s_ = -0.407, p < 0.05).

In total, 471 yeast isolates were obtained from CHROMagar Candida. These isolates were identified as belonging to 96 different species (Table I). The most frequently isolated species were *C. parapsilosis*, *Rhodotorula mucilaginosa*, *Meyerozyma guilliermondii*, and *M. caribbica*. Yeast occurrence was higher in the sand samples than in the seawater samples. Some opportunistic pathogenic species, such as *Wickerhamomyces anomalus*, *Lodderomyces elongisporus*, *Clavispora lusitaniae*, *Pichia kudriavzevii*, *Exophiala dermatitidis*, and *C. albicans*, were found only in the sand samples. Thirty-eight species isolated using CHROMagar Candida have been associated with clinical diseases in humans.

**TABLE I:** Yeast species number of positive samples and colony-forming unit (CFU)/100 mL for water samples and CFU/g for sand samples isolated from samples collected at recreational beaches in the states of Rio de Janeiro and Paraná, Brazil, using CHROMagar Candida

Yeast species	Ocurrence (nº of samples)	Rio de Janeiro**				Paraná**			
		Winter		Summer		Winter		Summer	
		Sand	Water	Sand	Water	Sand	Water	Sand	Water
Candida parapsilosis*(*C. albicans*/*Lodderomyces* clade)	52	15 (1.1 - 193.9)	9 (4.0 - 16.0)	3 (1.6 -8.7)	4 (4.0 - 8.0)	6 (0.8 - 6.9)	10 (4.0 - 36.0)	3 (1.8)	2 (4.0 - 36.0)
Rhodotorula mucilaginosa*	51	19 (0.8 - 101.6)	10 (4.0 - 28.0)		3 (8.0 - 52.0)	11 (0.9 - 12.9)	5 (4.0 - 12.0)	2 (0.9)	1 (4.0)
Meyerozyma guilliermondii*	32	11 (1.0 - 9.8)	3 (4.0)	3 (0.8 - 4.7)	1 (4.0)	3 (2.0 - 64.4)		5 (0.9 - 8.1)	6 (4.0 - 44.0)
Meyerozyma caribbica*	28	8 (0.9 - 51.8)	1 (4.0)	8 (0.8 - 8.5)	2 (4.0 - 8.0)	7 (0.8 - 83.7)	1 (4.0)		
Wickerhamomyces anomalus*	16	11 (1.0 - 10.4)		4 (0.8 - 2.4)		1 (1.0)			
Candida tropicalis* (*C. albicans*/*Lodderomyces* clade)	15	2 (1.2 - 155.7)		6 (0.8 - 12.7)	3 (4.0 - 12.0)	2 (0.8 - 1.7)		2 (9.0 - 52.2)	
*Candida intermedia* (*Metschnikowia* clade)	14	5 (1.2 - 20.7)	1 (32.0)			1 (1.0)	1 (4.0)	5 (0.9 - 5.4)	1 (4.0)
Kodamaea ohmeri*	14	8 (1.0 - 21.7)	2 (4.0)		1 (4.0)	3 (0.9 - 1.1)			
Candida metapsilosis* (*C. albicans*/*Lodderomyces* clade)	10	1 (1.0)		2 (0.8 - 3.1)		6 (0.9 - 1.0)	1 (8.0)		
Candida orthopsilosis* (*C. albicans*/*Lodderomyces* clade)	10	5 (1.2 - 18.7)	1 (8.0)	1 (0.8)	1 (4.0)	1 (0.8)	1 (4.0)		
Aureobasidium pullulans*	9	8 (1.0 - 6.2)				1 (1.7)			
Candida haemulonii*(*Clavispora* clade)	8		4 (4.0 - 56.0)		1 (4.0)	1 (0.8)	2 (4.0)		
*Debaryomyces nepalensis*	8	8 (1.0 - 113.1)							
*Papiliotrema aurea*	8	3 (1.0 - 12.0)				3 (0.9 - 15.2)	2 (4.0 - 16.0)		
*Rhodotorula diobovata*	8	5 (1.1 - 8.0)				3 (0.8 - 5.2)			
Lodderomyces elongisporus*	7	5 (1.2 - 18.9)		1 (0.8)				1 (0.9)	
Trichosporon asahii*	7	3 (1.2 - 13.8)	1 (4.0)			3 (0.8 - 1.0)			
*Candida pseudointermedia* (*Metschnikowia* clade)	6	4 (0.9 - 9.6)	2 (4.0)						
*Candida pseudolambica* (*Pichia* clade)	6	1 (16.0)				2 (1.0 - 3.4)	2 (4.0)		1 (4.0)
Clavispora lusitaniae*	6	2 (1.2 - 17.2)		3 (0.8 - 1.6)		1 (0.9)			
Pichia kudriavzevii*	6	3 (1.0 - 48.0)		2 (1.6 - 6.3)		1 (1.1)			
*Pseudozyma hubeiensis*	6	3 (0.8 - 1.2)	1 (4.0)			1 (0.9)	1 (4.0)		
Diutina catenulata*	5	3 (2 - 5.8)	1 (4.0)			1 (0.9)			
Papiliotrema laurentii*	5	3 (0.9 - 13.0)				1 (1.2)		1 (1.8)	
*Rhodotorula paludigena*	5	2 (1.0 - 1.1)	1 (4.0)			2 (0.9 - 13.5)			
Trichosporon faecale*	5	1 (1.0)		2 (0.8 - 2.4)				1 (5.4)	1 (4.0)
*Candida akabanensis* (*Clavispora* clade)	4	3 (1.0 - 4.8)							1 (12.0)
Cutaneotrichosporon dermatis*	4	1 (3.6)			1 (4.0)		1 (4.0)	1 (0.9)	
*Hanseniaspora uvarum*	4	2 (0.9 - 8.8)	1 (8.0)					1 (11.7)	
*Papiliotrema flavescens*	4	1 (1.1)				2 (1.2 - 1.7)		1 (0.9)	
*Wickerhamomyces sydowiorum*	4	2 (0.9 - 1.0)					2 (4.0 - 12.0)		
*Saturnispora silvae*	3	1 (15.2)					1 (24.0)		1 (0.9)
*Candida spencermartinsiae* (*Yamadazyma* clade)	3		1 (8.0)					2 (0.9 - 2.7)	
*Cutaneotrichosporon debeurmannianum*	3		2 (4.0)		1 (4.0)				
Debaryomyces hansenii*	3					1 (0.8)	2 (8.0)		
Exophiala dermatitidis*	3	2 (1.0 - 1.2)		1 (1.0)					
*Nakazawaea siamensis*	3	1 (2.1)				1 (0.8)	1 (4.0)		
Torulaspora delbruecki*	3	2 (4.4 - 6.4)							1 (4.0)
Trichosporon coremiiforme*	3	3 (1.2 - 4.8)							
*Ustilago spermophora*	3					3 (1.1 - 8.9)			
Yarrowia lipolytica*	3					3 (1.1 - 3.0)			
Apiotrichum montevideense*	2	1 (2.0)					1 (4.0)		
Candida albicans* (*C. albicans*/*Lodderomyces* clade)	2	1 (1.0)				1 (0.9)			
*Candida conglobata* (*Yamadazyma* clade)	2	1 (0.8)						1 (34.2)	
Candida duobushaemulonii* (*Clavispora* clade)	2		2 (60.0)						
*Candida natalensis*	2	1 (1.6)		1 (1.1)					
Diutina neorugosa*	2			2 (1.1)					
*Meyerozyma neustonensis*	2			2 (1.1 - 24.1)					
*Candida norvegica* (*Barnettozyma* clade)	2							2 (2.7 - 3.6)	
Wickerhamiella pararugosa*	2			1 (0.8)	1 (4.0)				
*Candida phangngensis* (*Yarrowia* clade)	2						1 (4.0)	1 (1.8)	
*Wikerhamiella sorboxylosa*	2	1 (8.0)	1 (28.0)						
*Candida suratensis* (*Clavispora* clade)	2	2 (2.9 - 7.3)							
*Cutaneotrichosporon terricola*	2			1 (0.8)				1 (5.4)	
*Hanseniaspora opuntiae*	2					1 (3.3)	1 (4.0)		
*Kazachstania exigua*	2		1 (36.0)				1 (22.0)		
Wickerhamomyces onychis*	2		2 (4.0)						
*Rhodotorula taiwanensis*	2					2 (2.0 - 3.3)			
*Wickerhamiella* sp. 1	2	1 (0.8)	1 (12.0)						
*Wickerhamiella* sp. 2	2	2 (1.0 - 2.4)							
*Apiotrichum cacaoliposimilis*	1			1 (0.8)					
*Barnettozyma californica*	1					1 (1.1)			
*Candida blattae* (*Clavispora* clade)	1	1 (1.2)							
Candida glabrata* (*Nakaseomyces* clade)	1		1 (8.0)						
*Kazachstania humilis*	1						1 (4.0)		
*Candida mengyuniae* (*Cyberlindnera* clade)	1				1 (4.0)				
*Candida michaelii* (*Yamadazyma* clade)	1		1 (4.0)						
Diutina rugosa*	1				1 (4.0)				
Cystobasidium minutum*	1			1 (1.0)					
Cyberlindnera fabianii*	1							1 (11.7)	
Debaryomyces fabryi*	1					1 (2.0)			
*Hannaella luteola*	1					1 (1.7)			
*Hanseniaspora occidentalis*	1						1 (4.0)		
*Pichia terricola*	1					1 (3.3)			
Kluyveromyces marxianus*	1			1 (0.8)					
*Kodamaea* sp.	1	1 (2.2)							
*Lachancea kluyveri*	1						1 (4.0)		
*Metschnikowia* sp. 1	1	1 (10.8)							
*Metschnikowia* sp. 2	1						1 (16.0)		
*Naganishia liquefaciens*	1		1 (8.0)						
*Papiliotrema rajasthanensis*	1					1 (0.8)			
*Pichia kluyveri*	1						1 (8.0)		
*Pichia norvegensis*	1					1 (1.0)			
*Rhynchogastrema complexa*	1	1 (2.2)							
*Sporidiobolus pararoseus*	1					1 (0.9)			
*Sporidiobolus carnicolor*	1					1 (0.8)			
*Sporidiobolus japonicus*	1					1 (0.9)			
*Sporopachydermia lactativora*	1				1 (8.0)				
Trichosporon inkin*	1				1 (4.0)				
Trichosporon japonicum*	1		1 (4.0)						
*Yamadazyma barbieri*	1				1 (4.0)				
*Yamadazyma* sp. 1	1								1 (2.7)
*Wickerhamiella* sp. 3	1				1 (4.0)				
*Wickerhamiella* sp. 4	1						1 (16.0)		
*Wickerhamomyces* sp.	1						1 (4.0)		
*Zygoascus* sp.	1		1 (4.0)						

*: yeast species reported as opportunistic pathogens; **: the first number represents the number of positive samples and the numbers in brackets represent the range of density for that sample.

From mCA agar, 74 yeasts, belonging to 26 different species, were isolated (Table II). In this medium, *M. guilliermondii*, *C. tropicalis*, and *M. caribbica* were the most frequently recovered yeasts. Some species, such as *Yarrowia lipolytica* and *E. spinifera*, were isolated only from sand samples. Two isolates of *C. albicans* were recovered from seawater samples, and one from sand samples using this culture medium. Ten species isolated on mCA agar are regarded as opportunistic pathogens.

**TABLE II:** Yeast species number of positive samples and colony-forming unit (CFU)/100 mL for water samples and CFU/g for sand samples isolated from samples collected at recreational beaches in the states of Rio de Janeiro and Paraná, Brazil, using mCA agar

Yeast species	Ocurrence (nº of samples)	Rio de Janeiro**				Paraná**			
		Winter		Summer		Winter		Summer	
		Sand	Water	Sand	Water	Sand	Water	Sand	Water
Meyerozyma guilliermondii*	15	12 (0.8 - 44.6)		1 (0.8)		1 (2.5)		1 (63.0)	
Candida tropicalis* (*C. albicans*/*Lodderomyces* clade)	13	2 (1.2 - 2.1)		3 (0.8 - 3.9)	2 (4.0)	2 (0.9 - 3.4)		3 (0.9 - 38.7)	1 (4.0)
Meyerozyma caribbica*	8	1 (3.6)		4 (0.8 - 8.7)	1 (4.0)	2 (0.9)			
Yarrowia lipolytica*	5	3 (2.1 - 13.8)				2 (1.0 - 3.4)			
*Candida cylindracea* (*Ogataea* clade)	4	1 (1.0)	1 (8.0)			1 (7.7)	1 (4.0)		
Candida albicans* (*C. albicans*/*Lodderomyces* clade)	3	1 (1.0)					1 (4.0)		1 (4.0)
Lodderomyces elongisporus*	3	1 (1.0)			1 (4.0)	1 (0.9)			
*Candida phangnensis* (*Yarrowia* clade)	2					1 (1.7)		1 (4.5)	
*Candida* sp. (*Nakaseomyces* clade)	2								2 (4.0)
Exophiala spinifera*	2					2 (1.7)			
Hortaea werneckii*	2					1 (3.7)	1 (4.0)		
*Candida blankii*	1			1 (0.8)					
*Candida boidinii* (*Ogataea* clade)	1					1 (0.9)			
*Wickerhamiella infanticola*	1						1 (1.0)		
*Candida nonsorbophila*	1					1 (0.9)			
*Candida polysorbophila* (*Zygoascus* clade)	1					1 (2.2)			
*Wickerhamiella sorbophila*	1						1 (16.0)		
*Candida viswanathii* (*C. albicans*/*Lodderomyces* clade)	1							1 (0.9)	
*Exophiala alcalophila*	1					1 (0.9)			
*Hanseniaspora* sp. 1	1						1 (12.0)		
*Hanseniaspora* sp. 2	1								1 (20.0)
*Hanseniaspora uvarum*	1					1 (1.1)			
*Rhodosporidiobolus ruineniae*	1							1 (0.9)	
*Rhodotorula diobovata*	1					1 (0.8)			
Trichosporon asahii*	1							1 (2.7)	
Wickerhamomyces onychis*	1					1 (0.9)			

*: yeast species reported as opportunistic pathogens; **: the first number represents the number of positive samples and the numbers in brackets represent the range of density for that sample.

One-hundred and forty-four yeast isolates from both culture media, whose species were reported as opportunistic pathogens, were tested to determine their MICs to fluconazole, itraconazole, and amphotericin B (Table III). Sixteen strains (11%) were resistant to fluconazole, 19 (13%) to itraconazole, and 29 (20%) to amphotericin B. Furthermore, 24 (17%) strains were susceptible to fluconazole and 49 (34%) to itraconazole in a dose-dependent manner. Among the five *C. albicans* isolates tested, two were resistant to itraconazole, with one of the two also being resistant to fluconazole. All five *C. albicans* isolates were susceptible to amphotericin B. The reference strains of *C. albicans*, SC5314 and ATCC18804, were susceptible to all of the antifungals tested. Two yeast strains, *C. haemulonii* UFMG-CM-Y4456 and *C. tropicalis* UFMG-CM-Y4046, showed resistance to the three antifungals tested. The same yeasts that were subjected to the antifungal susceptibility testing were also evaluated for their proteinase activity (Table III). Overall, 57 yeast isolates (40%) presented halo formation on BSA medium and were considered positive. *C. albicans* (five isolates), *K. ohmeri* (five isolates), and *P. kudriavzevii* (three isolates) showed proteinase production for all tested isolates.

**TABLE III:** Minimum inhibitory concentration (*μg/mL*) of fluconazole, itraconazole, and amphotericin B, proteinase activity, and adhesion to buccal epithelial cells of yeasts isolated from sand and water samples collected at recreational beaches in the states of Rio de Janeiro and Paraná, Brazil

Yeast species	Nº of isolates tested	Fluconazol (µg/mL)		Itraconazol (µg/mL)		Anfotericina B (µg/mL)		Proteinase activity			Adhesion	
		Range	S-DD-R*	Range	S-DD-R	Range	S-R	-	1+	2+	n	Yeasts/50 BECs
*Aureobasidium pullulans*	4	16.0-64.0	0-3-1	0.062-0.5	2-2-0	0.125-1.0	4-0	3	1	0	3	6-7
*Candida albicans*	5	0.25->64.0	4-0-1	0.031->16.0	3-0-2	0.5-1.0	5-0	0	0	5	5	95-198
*Candida cylindracea*	4	8.0-16.0	1-3-0	0.125-0.25	3-1-0	2.0-8.0	0-4	1	1	2	4	9-47
*Candida duobushhaemulonii*	1	8.0	1-0-0	0.125	1-0-0	2.0	0-1	1	0	0	1	24
*Candida glabrata*	1	2.0	0-1-0	0.062	1-0-0	0.125	1-0	0	0	1	0	ND
*Candida haemulonii*	2	8.0-64.0	1-0-1	0.5-16.0	0-1-1	1.0-2.0	1-1	1	0	1	2	68-73
*Candida metapsilosis*	1	1.0	1-0-0	0.062	1-0-0	0.5	1-0	1	0	0	0	ND
*Candida orthopsilosis*	6	0.5-4.0	6-0-0	0.031-0.125	6-0-0	0.015-0.5	6-0	4	1	1	1	2
*Candida parapsilosis*	10	0.25-1.0	10-0-0	0.031-0.125	10-0-0	0.031-1.0	10-0	6	3	1	4	2-112
*Candida tropicalis*	16	0.25->64.0	5-2-9	0.031->16.0	6-0-10	0.062-2.0	13-3	11	3	2	14	6-136
*Candida viswanathii*	1	8.0	1-0-0	0.25	0-1-0	1.0	1-0	0	1	0	1	11
*Clavispora lusitaniae*	3	0.5-2.0	3-0-0	0.125-0.5	1-2-0	0.125-1.0	3-0	2	0	1	2	1-4
*Cutaneotrichosporon dermatis*	2	2.0	2-0-0	0.0625	2-0-0	0.062-0.125	2-0	2	0	0	0	ND
*Debarymoyces fabryi*	1	0.25	1-0-0	0.25	0-1-0	2.0	0-1	1	0	0	1	13
*Debaryomyces hansenii*	1	0.5	1-0-0	0.125	1-0-0	2.0	0-1	0	0	1	1	18
*Diutina catenulata*	3	0.5-4.0	3-0-0	0.015-0.031	3-0-0	0.062-0.5	3-0	1	0	2	0	ND
*Diutina neorugosa*	2	4.0	2-0-0	0.125	2-0-0	0.5-1.0	2-0	2	0	0	0	ND
*Exophiala dermatitidis*	1	4.0	1-0-0	0.03125	1-0-0	0.25	1-0	1	0	0	0	ND
*Exophiala spinifera*	2	32.0	0-2-0	0.125	2-0-0	0.031-0.25	2-0	2	0	0	2	9-21
*Hortaea werneckii*	1	16.0	0-1-0	0.031	1-0-0	0.062	1-0	1	0	0	1	31
*Kodamaea ohmeri*	5	2.0-8.0	5-0-0	0.125-0.25	3-2-0	0.5-8.0	4-1	0	0	5	4	135-286
*Lodderomyces elongisporus*	8	0.125-2.0	8-0-0	0.031-0.5	7-1-0	0.015-2.0	7-0-1	6	1	1	8	2-52
*Meyerozyma caribbica*	7	4.0-16.0	5-2-0	0.25-1.0	0-6-1	0.5-2.0	6-1	6	0	1	7	6-44
*Meyerozyma guilliermondii*	17	2.0-16.0	12-5-0	0.125-2.0	1-14-2	0.25-2.0	16-1	8	1	8	16	4-113
*Papiliotrema laurentii*	3	2.0-4.0	3-0-0	0.031-0.125	3-0-0	0.015-0.25	3-0	2	0	1	0	ND
*Pichia kudriavzevii*	3	6.0-16.0	2-1-0	0.062-0.25	2-1-0	0.25-2.0	2-1	0	0	3	2	2-17
*Rhodotorula mucilaginosa*	9	0.125-64.0	1-4-4	0.031-2.0	2-4-3	0.015-1.0	9-0	9	0	0	8	1-49
*Trichosporon asahii*	8	0.5-4.0	8-0-0	0.125-0.25	4-4-0	0.5-16.0	2-6	8	0	0	8	2-80
*Trichosporon inkin*	1	1.0	1-0-0	0.031	1-0-0	0.125	1-0	1	0	0	0	ND
*Wickerhamiella infanticola*	1	4.0	1-0-0	0.25	0-1-0	0.5	1-0	0	0	1	1	6
*Wickerhamiella pararugosa*	1	0.25	1-0-0	0.031	1-0-0	0.062	1-0	1	0	0	0	ND
*Wickerhamomyces anomalus*	5	2.0-4.0	5-0-0	0.031-0.25	2-3-0	0.015-0.5	5-0	2	0	3	4	8-27
*Wickerhamomyces onychis*	2	0.25-2.0	2-0-0	0.031-0.125	2-0-0	0.062-2.0	1-1	2	0	0	1	4
*Yarrowia lipolytica*	7	0.25-8.0	7-0-0	0.062-0.5	2-5-0	1.0-2.0	1-6	2	1	4	7	2-73
Control strains												
*Candida albicans* ATCC18804	1	4	1-0-0	0.062	1-0-0	0.062	1-0	0	1	0	1	33
*Candida albicans* SC5314	1	8	1-0-0	0.5	1-0-0	0.5	1-0	1	0	0	1	59

*: S - number of susceptible yeast isolates; DD - number of dose-dependent susceptible yeast isolates; R - number of resistant yeast isolates. Susceptible, dose-dependent or resistant classification was based on the values recommended by the CLSI (2008, 2012), even though such documents were especially developed for *Candida* spp. and *Cryptococcus neoformans*. ND: not determined.

Two reference strains of *C. albicans* and 110 yeast isolates obtained in this study were evaluated in relation to their ability to adhere to BECs (Table III). Adhesion varied considerably, even between strains belonging to the same species. *K. ohmeri* exhibited adhesion rates ranging between 135 and 286 yeast cells·(50 BECs)^-1^. Strains of *C. albicans* recovered from water and sand samples also presented adhesion rates between 95 and 198 yeast cells·(50 BECs)^-1^. Two strains of *C. haemulonii* exhibited adhesion rates between 68 and 73 yeast cells·(50 BECs)^-1^. Other species too, such as *C. parapsilosis*, *C. tropicalis*, *M. guilliermondii*, and *T. asahii*, had strains with a high yeast cell per 50 BECs count.

**Fig. 1:**
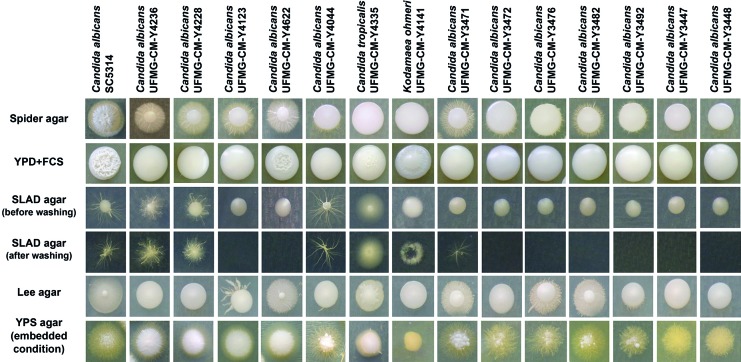
colony morphology of *Candida albicans*, *C. tropicalis* and *Kodamaea ohmeri* strains on different hypha-inducing media*.*

**Fig. 2:**
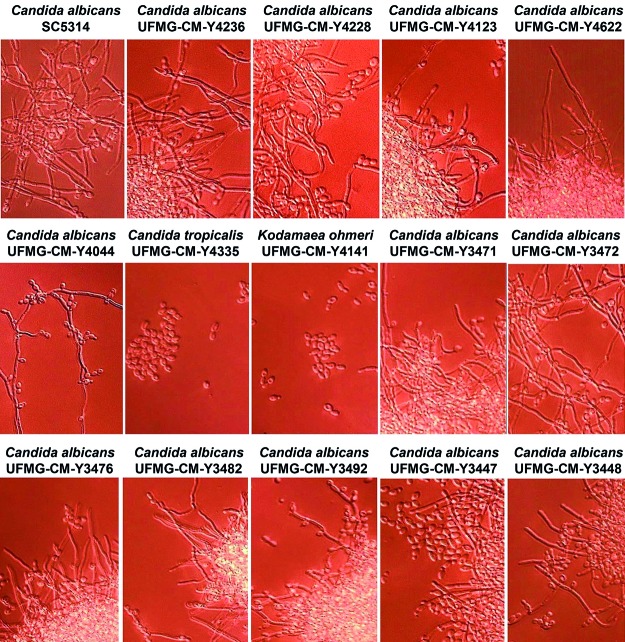
*Candida albicans*, *C. tropicalis* and *Kodamaea ohmeri* strains growing in a liquid yeast extract-peptone-dextrose (YPD) medium supplemented with foetal calf serum 10% and visualised through an Olympus FV1000 confocal microscope (40x).

**Fig. 3:**
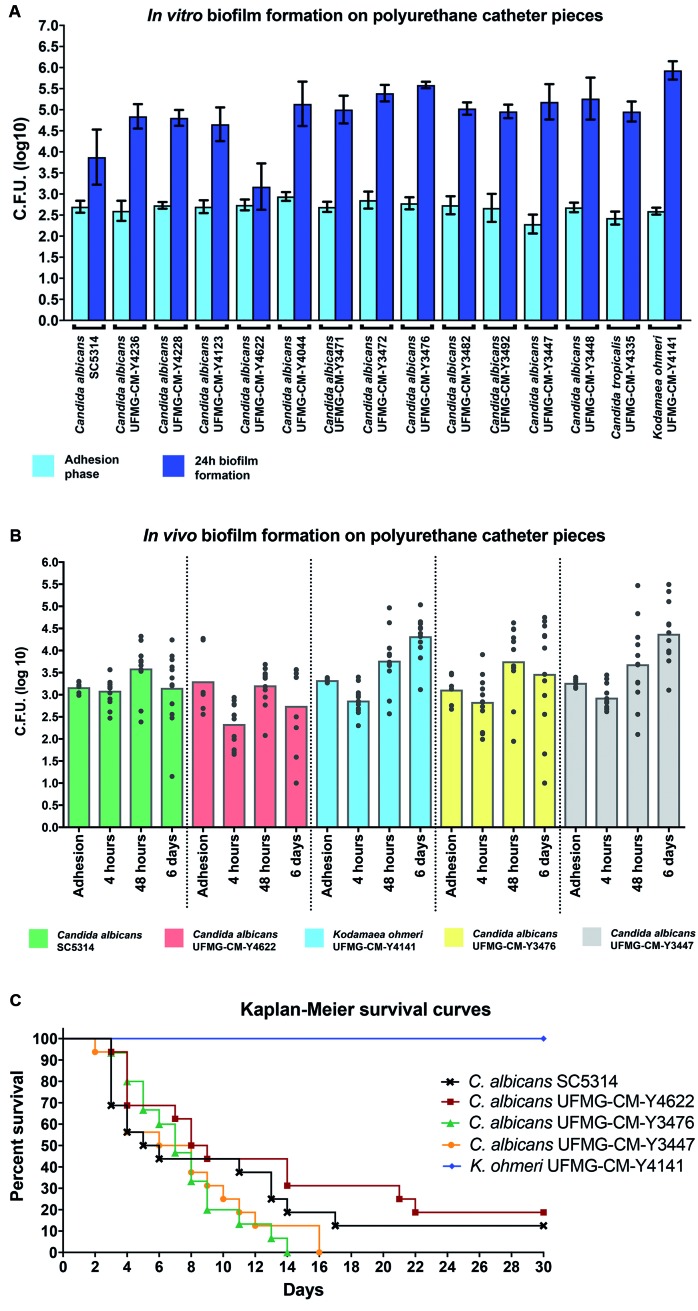
biofilm formation and *in vivo* virulence assays. (A) *In vitro* biofilm formation. Bars indicate the mean values for log10 numbers of colony-forming unit (CFU) of *Candida albicans*, *C. tropicalis* and *Kodamaea ohmeri* strains recovered from each catheter piece and respective standard deviations. (B) *In vivo* biofilm formation. Full circles represent the log10 numbers of CFU of *C. albicans* and *K. ohmeri* strains retrieved from each catheter piece. Bars indicate the mean values for log10 numbers of CFU obtained per catheter piece. (C) Kaplan-Meier survival curves of BALB/c mice after infection with *C. albicans* and *K. ohmeri* strains.

As previously described in the material and methods section, the five *C. albicans* strains isolated from the beaches, the seven other *C. albicans* strains isolated from the lakes, and the reference strain *C. albicans* SC5314 were evaluated for additional virulence factors (morphogenesis ability, biofilm formation *in vitro* and *in vivo*, and virulence *in vivo*). Additionally, two yeast strains belonging to different species were included in these analyses: *K. ohmeri* UFMG-CM-Y4141, which presented a positive proteinase activity and adhesion to BECs up to 286 yeast cells·(50 BECs)^-1^, and *C. tropicalis* UFMG-CM-Y4335, which was resistant to fluconazole and itraconazole, showed proteinase production, and adhesion to BECs. First, the selected strains were tested with respect to their morphogenesis ability under different hypha-inducing conditions. A wide variety of response profiles to the different media were observed (Figs 1-2). In general, environmental strains developed colonies with a filamentous appearance in at least three of the five hyphae-inducing media. Exceptions were *K. ohmeri* and two *C. albicans* strains (strains UFMG-CM-Y3447 and UFMG-CM-Y3448) recovered from the lakes as they developed filamentous appearance in only two solid media. Microscopically, all yeasts grown in YPD broth supplemented with foetal calf serum presented hyphae or pseudohyphae or both, except the *C. tropicalis* and *K. ohmeri* strains. Then, the selected strains were subjected to *in vitro* biofilm formation assays (Fig. 3A). In the adhesion phase, two strains of *C. albicans*, UFMG-CM-Y4044 and UFMG-CM-Y3472, showed a significantly higher number of cells adhering to catheters than the reference strain (U-test, p < 0.05). Furthermore, *C. albicans* UFMG-CM-Y3447 and *C. tropicalis* UFMG-CM-Y4335 exhibited less significant adhesion than the reference strain (U-test, p < 0.05). In the biofilm formation tests, nearly all the yeast strains presented significantly higher numbers of CFU recovered from catheter pieces than *C. albicans* SC5314 (U-test, p < 0.05). *C. albicans* UFMG-CM-Y4622 was the only one with a significantly lower number of CFU obtained from catheter pieces (U-test, p < 0.05).

*In vivo* biofilm formation assays were performed for *C. albicans* SC5314 and other four yeast strains: *K. ohmeri* UFMG-CM-Y4141, *C. albicans* UFMG-CM-Y3476, *C. albicans* UFMG-CM-Y3447, and *C. albicans* UFMG-CM-Y4622 (Fig. 3B). The first three environmental strains were chosen because of their *in vitro* biofilm formation and the latter strain was selected because it presented the lowest biofilm formation in the same experiments. Initially, yeasts adhered similarly to catheters (mean = 3.24 ± 0.10 log10 CFU/catheter) and there were no significant differences among them and the wild type strain (U-test, p > 0.05). Four hours after device implantation, the numbers of CFU retrieved from catheter fragments declined slightly for all the strains tested (mean = 2.81 ± 0.06 log10 CFU/catheter), but this decrease was not statistically significant (U-test, p > 0.05). *C. albicans* UFMG-CM-Y4622 was the only strain that presented a significant lower number of CFU recovered from the polyurethane devices compared to *C. albicans* SC5314 (U-test, p < 0.001). There were no significant differences in the biofilm formation among the other strains (U-test, p > 0.05). All yeasts tested showed biofilm development (mean = 3.60 ± 0.09 log10 CFU/catheter) 48 h after catheter implantation. At this point, biofilms were in the maturation phase and CFU counts increased significantly (U-test, p ≤ 0.01). Once again, *C. albicans* UFMG-CM-Y4622 was the only strain with a significant lower number of CFU retrieved from catheter pieces compared to *C. albicans* SC5314 (U-test, p < 0.05). Six days after implantation, although not statistically significant, there was a slight decrease in the CFU counts for *C. albicans* SC5314, *C. albicans* UFMG-CM-Y4622, and *C. albicans* UFMG-CM-Y3476 (U-test, p > 0.05). A significant increase in the number of CFU recovered from catheter fragments for *C. albicans* UFMG-CM-Y3447 and for *K. ohmeri* UFMG-CM-Y4141 (U-test, p < 0.05) was observed. At the end of the tests, *C. albicans* UFMG-CM-Y3447 and *K. ohmeri* UFMG-CM-Y4141 developed significantly more abundant biofilms than the wild type strain (U-test, p < 0.05).

Finally, the same yeast strains subjected to the *in vivo* biofilm formation tests were also evaluated for their virulence in a murine model. BALB/c mice were challenged with the *C. albicans* and *K. ohmeri* strains and monitored for up to 30 days. All *C. albicans* strains were able to cause disease in the mice. In the days following infection, animals showed ruffled hair and gradual weight loss. Within two weeks, all mice inoculated with the reference strain and *C. albicans* UFMG-CM-Y3447 had died. Most mice inoculated with the other *C. albicans* strains also died in this period. Mice challenged with the *K. ohmeri* UFMG-CM-Y4141 presented no signs of disease and remained alive until the end of the experiment (Fig. 3C).

## DISCUSSION

According to the Brazilian legislation, seawater is considered inappropriate for primary contact recreation when the last collected sample has an *E. coli* density above 2,000 MPN·(100 mL)^-1^.^19^ According to this, eight samples from Paraná (22%) and 19 samples from Rio de Janeiro (53%) in this study could be classified as inappropriate [Supplementary data (Tables I-II)]. Rio de Janeiro probably had more samples with higher *E. coli* densities because it is a bigger city, with a much larger population, a higher level of urbanisation, and because it is visited by a high number of tourists, mainly during the summer. These features contribute to an elevated level of organic pollution resulting from the anthropic pressure and, consequently, to water contamination. It has already been observed a strong positive association between the presence of conventional bacterial indicators in marine waters and the incidence of gastrointestinal illnesses among bathers.^20^

In Brazil, there is no federal legislation that establishes microbiological parameters to evaluate the quality of beach sand. However, in the city of Rio de Janeiro, a municipal resolution exists, that provides some microbiological thresholds. According to this resolution, a sand sample is classified as not recommended for recreational purposes when it has above 38 MPN·g^-1^ of *E. coli*.^21^ In this context, 14 samples from Rio de Janeiro (19%) were above the threshold [Supplementary data (Table I)]. In the Paraná state, there is no local legislation for sand, but if the same limit used in Rio de Janeiro is applied, 12 samples (17%) would be above the threshold [Supplementary data (Table II)]. Regarding recreational activities on beaches, the health risks associated with sand contact remain unclear. However, Heaney and colleagues^2^ reported a positive correlation between activities in the sand (i.e., digging and burying) and the incidence of gastroenteritis, with burying in the sand being more closely associated with infections.

In our study, there was a greater number of wet sand samples with high *E. coli* counts than of dry sand samples with high *E. coli* counts. Some features, such as the increased protection from sunlight, buffered temperatures, and higher nutrient availability, can favor the persistence, survival, and regrowth of *E. coli* in the sand.^3^

In Brazil, there are no established standards for yeast levels in beach seawater and sand. In our study, four seawater samples had yeast counts above 100 CFU·(100 mL)^-1^, which is considerated high: two samples collected at point #3 on Copacabana beach (Rio de Janeiro), one sample from Matinhos Central beach (Paraná), and one from Mansa beach (Paraná). Samples from Paraná with high counts were collected in front of the drainage systems, which are expected to be more contaminated. However, in the samples from Rio de Janeiro, high yeast counts in the water at Copacabana beach coincided with high yeast counts in the sand samples collected at the same transects. Among the sand samples, yeast populations varied drastically, indicating a heterogeneous distribution, ultimately making interpretation difficult. Vogel et al.^10^ evaluated the prevalence of yeasts in the sand at three bathing beaches in South Florida (USA) and found such heterogeneity as well. They suggested that these microorganisms primarily live in the sand, later serving as contamination sources for the water column. The prevalent yeast species isolated in our study are associated with organic pollution of human origin, and probably, are resulted from inputs of terrestrial sources as sewages.

In the present study, similar to the results by Vogel et al.,^10^ the supralittoral zone harbored the highest yeast counts, which is in accordance with the higher number of yeasts found in dry sand than in wet sand. Apparently, the main sources of contamination of dry sand with these microorganisms are inputs from soil, runoff, and beach users. It has already been proposed the dry sand as an ideal matrix for fungal analysis, because fungi can survive in this environment better than enteric bacteria.^1^

While there were no significant correlations between faecal indicator bacteria and yeast densities in either the water or sand samples, other authors observed different results. A study performed in 33 beaches in Portugal found that, although yeasts correlated positively with coliforms in sand samples, they did not correlate with *E. coli* and intestinal enterococci.^1^ In contrast, a study carried out on a subtropical beach in Miami (USA) found a significant positive correlation between red and white yeasts and faecal coliforms in sand samples.^9^ Because the correlation between fungi and traditional indicators is clearly variable, some studies support the adoption of additional microbiological indicators for assessing the quality of recreational waters and sands.^1^

The yeast species most frequently recovered from CHROMagar Candida and mCA agar have been shown to have some clinical importance. Besides being a common human commensal, *C. parapsilosis* has been sporadically recovered from a variety of substrates and localities such as domestic animals, insects, soil, and marine environments. Over the past decade, the incidence of *C. parapsilosis* in nosocomial infections has increased drastically, and it has also been reported that *C. parapsilosis* is more frequently associated with neonatal and pediatric patients with low birth weights, parental malnutrition, and hematological malignancies.^19^ ^,^ ^22^ *Rh. mucilaginosa* is widely distributed in nature, but, of late, it has also emerged as an opportunistic pathogen, being related to many cases of fungemia associated with catheters, endocarditis, peritonitis, meningitis, and endophthalmitis, mainly in immunocompromised patients.^23^ *M. guilliermondii* is widely distributed in natural environments, and it is also a part of the saprophytic human skin and mucosal microflora. It can lead to severe opportunistic fungal infections such as candidemia.^22^ *C. tropicalis* has been isolated from different substrates such as fruit, flowers, soil, water, and clinical specimens^19^ ^,^ ^22^ and has been recognised as an increasing cause of bloodstream infections outside the United States, particularly in South America and Asia.^24^ *C. tropicalis* and *Rh. mucilaginosa* were the most abundant species recovered from sand obtained from three beaches in South Florida (USA).^10^ In addition, in the same study, 12 other species in common with the results of our study were isolated: *C. albicans*, *Clavispora lusitaniae*, *Diutina catenulata*, *K. ohmeri*, *P. kudriavzevii*, *Rh. paludigena*, *Torulaspora delbrueckii*, *Trichosporon asahii*, *Tr. coremiiforme*, *W. anomalus*, *W. onychis*, and *Y. lipolytica*.

Although *C. albicans* was isolated from only a few samples (five), it is important to emphasise its occurrence because it remains the most common etiological agent of candidiasis. It can cause a variety of infections that range from superficial to life-threatening invasive candidiasis.^22^ Another study too have shown a low incidence of *C. albicans* retrieved from environmental samples: Sabino and colleagues^1^ detected it in only 0.8% of sand samples collected from 33 beaches in Portugal over a five-year period.

In the present study, at least 40 isolated yeast species, highlighted in Tables I-II, have already been reported as opportunistic pathogens.^22^ ^,^ ^25^ According to Sabino et al.^1^ although no correlation has been clearly demonstrated between health issues and the pathogenic fungi in beach sands and waters, it may be expected that bathers are at an increased risk of exposure through direct contact of their skin and mucous membranes with the sand and water or by inhalation of fungal propagules. Thus, the presence of a wide range of pathogenic microorganisms in the samples evaluated in this study suggests a potential threat for people who attend the beaches, especially for immunocompromised individuals.

A wide variety of yeasts resistant to the antifungal drugs tested were isolated from sand and water samples (Table III), the most notable being *C. haemulonii* UFMG-CM-Y4456 and *C. tropicalis* UFMG-CM-Y4046, which were resistant to all three of the tested antifungals. The *C. haemulonii* complex yeasts are emerging pathogens whose multi-resistant susceptibility profile represents a challenge to therapy.^26^ A study conducted in five hospitals in São Paulo (Brazil) demonstrated the prevalence of 0.3% of *C. haemulonii* among yeasts isolated from case-patients between January 2010 and March 2015. In general, *C. haemulonii* complex strains had high MICs for amphotericin B and fluconazole. Drug therapy failed in five of eight patients with candidemia: four were being treated with amphotericin B and one with fluconazole. Furthermore, the 30-day all-cause mortality rate among patients with candidemia was 50%.^26^ For *C. tropicalis*, it has been pointed out the increased resistance of clinical isolates to azoles, especially strains recovered in the Asia-Pacific region.^27^ Azole resistance is not uncommon for environmental isolates of such species. Zuza-Alves et al.,^28^ while testing 125 strains of *C. tropicalis* isolated from a sandy beach in Natal (Brazil), observed a high resistance to the three azoles tested: fluconazole, voriconazole, and itraconazole. These authors observed that two strains of *C. albicans* were resistant to itraconazole, while one was resistant to fluconazole. In addition, fifteen strains were resistant to all three azoles tested (24.2%), and some strains were resistant to amphotericin B as well (14 isolates; 22.6%). Apparently, environmental reservoirs of fungi resistant to antifungal drugs have been increasing. Authors from two studies conducted in Brazil also observed reduced antifungal susceptibility of yeasts recovered from sediment and water samples from lakes and rivers.^7^ They also proposed that the occurrence of yeasts resistant to common antifungal drugs could suggest potential health risks for people using aquatic environments that receive anthropogenic impacts for recreation.

The positive results of the adhesion to the host cell surface and the proteinase production tests may imply a higher pathogenic potential of the yeasts isolated from sand and water samples. Adhesion represents the first step in colonisation and subsequent infection. Proteinases are one of the major virulence factors of *C. albicans.* It is important to emphasise that many yeast isolates simultaneously showed poor antifungal susceptibility, high proteinase activity, and a high adhesion rate. For example, the *C. albicans* isolate UFMG-CM-Y4622 was resistant to both fluconazole and itraconazole, had a high proteinase production, and an adhesion rate of 115 yeast cells·(50 BECs)^-1^. In addition, the *C. haemulonii* strain UFMG-CM-Y4456 was resistant to all three antifungal agents and exhibited a high proteinase production and an adhesion rate of 68 yeast cells·(50 BECs)^-1^. Among all five *K. ohmeri* strains tested, two were susceptible to itraconazole in a dose-dependent manner, one was resistant to amphotericin B, and all were proteinase producers with high adhesion rates. Such results question the safety of leisure activities that expose bathers to these microorganisms.

Morphogenesis ability, observed for all 15 tested yeasts, is also an important virulence factor. Both yeast and hyphal growth forms are believed to play an important role in fungal infection. Although hyphae would be vital with respect to tissue damage and invasion, yeast cells would represent the form primarily involved in dissemination. A survey has shown that *C. albicans* strains that failed to form filaments in response to serum or other inducers of filamentous growth were suggested as avirulent in murine models, i.e., they were unable to cause disease in mice.^29^

Regarding the *in vitro* and *in vivo* biofilm formation assays, results showed the ability of environmental strains to form biofilms on polyurethane catheters and confirmed their potential to grow on implanted medical devices. Different strains of the same opportunistic *Candida* species can exhibit varied abilities to form a biofilm. There are few studies describing biofilm formation by *K. ohmeri*, but it is already known that the use of indwelling catheters or implants is an important risk factor associated with infections caused by such species.^30^ Interestingly, during the *in vivo* biofilm formation tests, CFU counts retrieved from catheter pieces slightly decreased after implantation and then, increased again. Řičicová et al.^18^ observed the same phenomenon during the development of this subcutaneous model of biofilm formation. The authors suggested that, possibly, in the first hours after implantation, cells detach more easily from catheters being removed during experimental procedure or do not adapt to the growth conditions inside the host.

Results from the *in vivo* virulence assay suggest that the environmental strains of *C. albicans* were as virulent as the reference strain SC5314, which was originally isolated from a blood culture from a patient with disseminated candidiasis. More studies are necessary to evaluate whether these microorganisms represent a potential risk of infection to those individuals who have direct contact with them during recreational activities. Besides the various virulence factors that were demonstrated in the previous experiments for *K. ohmeri* UFMG-CM-Y4141, this strain was avirulent in the mouse model adopted here. However, it is worth mentioning that, whereas *K. ohmeri* is known to cause diseases mainly in immunocompromised individuals or those with some underlying conditions,^30^ in the present study, it was tested on immunocompetent mice.

*In conclusion* - The occurrence of opportunistic yeast species in water and sand samples collected from six Brazilian beaches suggests a potential risk to the health of beach users, mainly because many isolates presented important virulence factors and poor susceptibility to common antifungal drugs. Furthermore, results from disseminated infection assays showed that yeasts isolated from environmental samples could retain their virulence and cause disease. In addition, sand was shown to harbor a high density of faecal indicator bacteria and yeasts, thus serving as a reservoir of these microorganisms. Therefore, it seems reasonable that sands need to be urgently included as part of beach monitoring programs.
