# ERRATUM: Occurrence, antifungal susceptibility, and virulence factors of opportunistic yeasts isolated from Brazilian beaches

**DOI:** 10.1590/0074-02760180566ER

**Published:** 2019-04-15

In the article “**Occurrence, antifungal susceptibility, and virulence factors of opportunistic yeasts isolated from Brazilian beaches”**, DOI number: 10.1590/0074-02760180566, published in *Mem Inst Oswaldo Cruz*, Rio de Janeiro, Vol. 114: e180566, 2019, on page 1: 

Where it reads:

Dijck PV

It should read:

Van Dijck P

