# Supplementary material for: Occurrence, antifungal susceptibility, and virulence factors of opportunistic yeasts isolated from Brazilian beaches
Source: Mem Inst Oswaldo Cruz. 2019 Mar 14;114:e180566. doi: 10.1590/0074-02760180566 (PMC6419412; doi:10.1590/0074-02760180566)
Supplement: Supplementary file 1 [file 1678-8060-mioc-114-e180566-s.pdf]

TABLE I

Yeast, total coliforms, and *Escherichia coli* densities in sand and water samples [colony-forming unit (CFU) or MPN/100 mL for water samples and CFU or MPN/g for sand samples] collected at recreational beaches in the state of Rio de Janeiro, Brazil

				Yeast (CFU/g or 100 mL)		Total coliforms (MPN/g or 100 mL)		*E. coli* (MPN/g or 100 mL)	
State	Beach	Transect*	Zone**	Winter	Summer	Winter	Summer	Winter	Summer
Rio de Janeiro	Ipanema 1	1	Supra	6.5	0.0	142.7	0.8	2.5	0.5
			Medio	25.6	0.0	20.5	3.2	< 0.16	3.0
			Infra	4.3	1.0	> 517.3	> 463.8	7.0	> 463.8
			Water	24.0	0.0	> 4839.2	> 4839.2	3465.8	> 4839.2
		2	Water	84.0	8.0	> 4839.2	> 4839.2	4839.2	1454.0
		3	Supra	0.9	5.7	0.2	0.7	0.2	< 0.2
			Medio	5.8	0.8	< 0.2	125.4	0.8	56.1
			Infra	2.1	0.0	171.6	> 481.0	6.0	308.8
			Water	0.0	20.0	2599.4	> 4839.2	689.6	> 4839.2
	Ipanema 2	1	Supra	64.0	142.4	379.4	0.3	270.0	< 0.2
			Medio	13.4	9.6	6.3	> 387.8	5.4	> 387.8
			Infra	1.2	2.5	61.4	102.6	3.8	38.2
			Water	8.0	4.0	1841.6	> 4839.2	581.8	1841.6
		2	Water	20.0	0.0	> 4839.2	> 4839.2	520.6	1960.8
		3	Supra	145.1	4.0	115.0	4.0	18.7	< 0.2
			Medio	15.0	0.8	2.2	4.1	< 0.2	0.6
			Infra	0.0	0.0	43.5	294.7	0.5	53.3
			Water	0.0	4.0	1454.0	> 4839.2	1034.4	1034.4
	Ipanema 3	1	Supra	133.2	80.0	8.9	25.9	0.2	0.2
			Medio	2.7	7.9	3.0	10.9	1.7	4.4
			Infra	0.0	0.9	155.3	202.2	22.6	28.1
			Water	4.0	12.0	1258.8	4839.2	67.4	2827.2
		2	Water	28.0	20.0	944.2	> 4839.2	296.6	2599.4
		3	Supra	244.6	0.8	> 501.7	0.3	3.9	< 0.2
			Medio	139.4	21.4	1.7	10.7	<0.2	3.2
			Infra	1.2	0.0	23.9	186.0	3.2	16.7
			Water	8.0	0.0	516.2	> 4839.2	61.0	1297.6

				Yeast (CFU/g or 100 mL)		Total coliforms (MPN/g or 100 mL)		*E. coli* (MPN/g or 100 mL)	
State	Beach	Transect*	Zone**	Winter	Summer	Winter	Summer	Winter	Summer
Rio de Janeiro	Copacabana 1	1	Supra	250.0	0.0	5.6	0.3	< 0.2	< 0.2
			Medio	56.8	1.6	0.2	148.0	< 0.2	78.5
			Infra	2.3	408.5	7.6	> 449.3	1.7	7.7
			Water	16.0	32.0	2406.6	> 4839.2	821.2	1659.4
		2	Water	56.0	28.0	> 4839.2	> 4839.2	> 4839.2	> 4839.2
		3	Supra	12.0	3.2	4.7	0.3	4.7	< 0.2
			Medio	0.8	4.0	< 0.2	64.8	< 0.2	45.1
			Infra	0.0	0.0	7.6	59.0	4.7	14.0
			Waterw	64.0	4.0	> 4839.2	> 4839.2	> 4839.2	> 4839.2
	Copacabana 2	1	Supra	124.4	0.0	5.1	14.1	0.2	< 0.2
			Medio	1.8	9.5	0.2	> 384.2	< 0.2	> 384.2
			Infra	15.6	2.8	38.7	44.0	13.0	17.7
			Water	36.0	8.0	> 4839.2	> 4839.2	3465.8	1841.6
		2	Water	12.0	24.0	4839.2	> 4839.2	2406.6	3972.6
		3	Supra	114.7	2.4	13.0	< 0.2	1.1	< 0.2
			Medio	2.8	0.0	< 0.2	39.7	0.4	22.7
			Infra	2.3	0.9	12.2	17.2	4.6	5.2
			Water	20.0	8.0	> 4839.2	> 4839.2	922.2	2406.6
	Copacabana 3	1	Supra	367.0	32.3	> 558.5	3.6	> 558.5	< 0.2
			Medio	24.7	0.0	1.2	251.2	11.3	28.8
			Infra	0.0	0.0	7.8	> 476.1	7.5	143.0
			Water	104.0	8.0	> 4839.2	> 4839.2	2.0	> 4839.2
		2	Water	36.0	20.0	> 4839.2	> 4839.2	3465.8	4839.2
		3	Supra	222.62	0.0	417.1	< 0.2	251.8	< 0.2
			Medio	5.3	0.0	430.7	8.0	> 430.7	0.8
			Infra	3.7	9.9	39.9	14.4	20.2	5.2
			Water	128.0	24.0	> 4839.2	> 4839.2	2406.6	4839.2

*: transect 1 - 50 m to the south of the drainage system of pluvial waters; transect 2 - in front of the drainage system; transect 3 - 50 m to the south of the drainage system. **: supra - supralittoral; medio - mediolittoral; infra - infralittoral.

CC BY

TABLE II

Yeast and *Escherichia coli* densities in sand and water samples (CFU or MPN/100 mL for water samples and CFU or MPN/g for sand samples) collected at recreational beaches in the state of Paraná, Brazil

				Yeast (CFU/g or 100 mL)		*E. coli* (MPN/g or 100 mL)	
**State**	**Beach**	**Transect***	**Zone****	**Winter**	**Summer**	**Winter**	**Summer**
**Paraná**	**Mansa**	**1**	Supra	5.8	22.5	0.2	2.2
			Medio	2.8	0.0	3.7	0.6
			Infra	4.4	34.2	0.7	0.4
			Water	20.00	60.0	2239.8	151.6
		**2**	Water	68.00	128.0	1740.8	98.8
		**3**	Supra	4.2	5.4	0.2	2.0
			Medio	5.9	5.4	0.2	0.1
			Infra	3.5	0.0	0.2	7.2
			Water	20.00	64.0	3106.2	61.2
	**Central**	**1**	Supra	45.6	8.1	262.1	0.1
			Medio	7.0	0.9	20.6	23.9
			Infra	15.6	1.8	12.0	54.7
			Water	16.0	24.0	3972.6	136.4
		**2**	Water	172.0	0.0	3465.8	249.6
		**3**	Supra	149.5	1.8	9.6	3.5
			Medio	9.0	4.5	483.4	36.6
			Infra	17.6	5.4	179.6	28.4
			Water	68.0	52.0	4839.2	1379.2
	**Flórida**	**1**	Supra	19.4	4.5	0.2	10.5
			Medio	8.2	28.8	45.4	19.0
			Infra	3.0	0.9	483.4	19.2
			Water	16.0	16.0	229.0	310.4
		**2**	Water	0.0	16.0	357.8	> 9678.4
		**3**	Supra	3.4	8.1	5.0	0.1
			Medio	1.7	0.9	0.2	0.1
			Infra	2.2	0.0	128.2	9.6
			Water	4.0	28.0	372.0	134.8
**Paraná**	**Leste**	**1**	Supra	8.7	18.0	0.2	0.1
			Medio	8.4	0.9	7.7	1.2
			Infra	0.0	10.8	1.3	0.3
			Water	4.0	0.0	1226.2	2595.2
		**2**	Water	0.0	0.0	615.2	2004.8
		**3**	Supra	6.1	0.0	0.2	ND
			Medio	0.0	0.0	0.2	5.1
			Infra	0.0	3.6	0.2	1.8
			Water	0.0	0.0	370.0	1740.8
	**Ipanema**	**1**	Supra	54.9	> 270.0	0.2	7.8
			Medio	5.6	17.1	85.8	1.2
			Infra	3.4	1.8	16.7	1.1
			Water	12.0	0.0	449.4	665.6
		**2**	Water	16.0	20.0	581.8	440.0
		**3**	Supra	8.5	133.2	0.2	0.6
			Medio	11.1	6.3	13.7	5.0
			Infra	1.2	2.7	12.5	3.0
			Water	4.0	36.0	186.6	688.8
	**Shangrilá**	**1**	Supra	0.0	6.3	0.7	0.5
			Medio	9.9	7.2	530.9	0.1
			Infra	2.2	1.8	74.4	12.0
			Water	0.0	4.0	296.4	1376.0
		**2**	Water	0.0	56.0	247.2	898.8
		**3**	Supra	83.9	0.0	0.2	0.1
			Medio	5.6	3.6	62.8	51.4
			Infra	1.2	1.8	6.7	9.3
			Water	4.0	48.0	323.2	688.8

*: transect 1 - 50 m to the south of the drainage system of pluvial waters; transect 2 - in front of the drainage system; transect 3 - 50 m to the south of the drainage system. **: supra - supralittoral; médio - mediolittoral; infra - infralittoral.
